# Tumor stromal vascular endothelial growth factor A is predictive of poor outcome in inflammatory breast cancer

**DOI:** 10.1186/1471-2407-12-298

**Published:** 2012-07-19

**Authors:** Hugo Arias-Pulido, Nabila Chaher, Yun Gong, Clifford Qualls, Jake Vargas, Melanie Royce

**Affiliations:** 1 Departments of Internal Medicine, The University of New Mexico Cancer Center, Albuquerque, NM, USA; 2 Department of Pathology, Centre Pierre et Marie Curie, 1, Avenue Battendier, Place May 1st, Algiers, Algeria; 3 Department of Pathology, The Morgan Welch Inflammatory Breast Cancer Research Program and Clinic, The University of Texas MD Anderson Cancer Center, Houston, TX, USA; 4 Mathematics and Statistics, The University of New Mexico Cancer Center, Albuquerque, NM, USA

## Abstract

**Background:**

Inflammatory breast cancer (IBC) is a highly angiogenic disease; thus, antiangiogenic therapy should result in a clinical response. However, clinical trials have demonstrated only modest responses, and the reasons for these outcomes remain unknown. Therefore, the purpose of this retrospective study was to determine the prognostic value of protein levels of vascular endothelial growth factor (VEGF-A), one of the main targets of antiangiogenic therapy, and its receptors (VEGF-R1 and -R2) in IBC tumor specimens.

**Patients and Methods:**

Specimens from IBC and normal breast tissues were obtained from Algerian patients. Tumor epithelial and stromal staining of VEGF-A, VEGF-R1, and VEGF-R2 was evaluated by immunohistochemical analysis in tumors and normal breast tissues; this expression was correlated with clinicopathological variables and breast cancer-specific survival (BCSS) and disease-free survival (DFS) duration.

**Results:**

From a set of 117 IBC samples, we evaluated 103 ductal IBC tissues and 25 normal specimens. Significantly lower epithelial VEGF-A immunostaining was found in IBC tumor cells than in normal breast tissues (P <0.01), cytoplasmic VEGF-R1 and nuclear VEGF-R2 levels were slightly higher, and cytoplasmic VEGF-R2 levels were significantly higher (P = 0.04). Sixty-two percent of IBC tumors had high stromal VEGF-A expression. In univariate analysis, stromal VEGF-A levels predicted BCSS and DFS in IBC patients with estrogen receptor-positive (P <0.01 for both), progesterone receptor-positive (P = 0.04 and P = 0.03), HER2+ (P = 0.04 and P = 0.03), and lymph node involvement (P <0.01 for both). Strikingly, in a multivariate analysis, tumor stromal VEGF-A was identified as an independent predictor of poor BCSS (hazard ratio [HR]: 5.0; 95% CI: 2.0-12.3; P <0.01) and DFS (HR: 4.2; 95% CI: 1.7-10.3; P <0.01).

**Conclusions:**

To our knowledge, this is the first study to demonstrate that tumor stromal VEGF-A expression is a valuable prognostic indicator of BCSS and DFS at diagnosis and can therefore be used to stratify IBC patients into low-risk and high-risk groups for death and relapses. High levels of tumor stromal VEGF-A may be useful for identifying IBC patients who will benefit from anti-angiogenic treatment.

## Background

Inflammatory breast cancer (IBC) is a rare but highly aggressive and lethal form of locally advanced breast cancer with clinical signs that mimic an inflammatory process, such as diffuse breast erythema, peau d’orange, skin induration, and warmth. Tumor emboli are often identified in the dermal lymphatics, although the emboli are not always seen on skin biopsy [1,2]. Furthermore, the high expression levels of angiogenic [3-6], lymphangiogenic [3,7], and vasculogenic mimicry factors [4,8,9] observed in IBC specimens is considered critical to IBC’s metastatic behavior [10,11].

Vascular endothelial growth factor-A (VEGF-A), one of the most potent promoters of angiogenesis and lymphangiogenesis, is a secreted ligand with specific receptors (VEGF-R1 and -R2) that are expressed principally by angioblasts and endothelial cells; it is involved in endothelial cell growth, motility, and blood vessel permeability [12,13]. Abnormal VEGF-A, VEGF-R1, and VEGF-R2 levels have been observed in various cancers, including IBC [3,6,14].

Given IBC’s highly angiogenic features, anti-angiogenic agents that target VEGF-A and VEGF-R2 have been evaluated in clinical trials [15-19]. Although complete pathological responses have been rare, a direct inhibitory effect on angiogenic parameters has been observed: specifically, 1) VEGF-A expression levels in tumor cells at baseline were higher in responders than in non-responders [16,17]; 2) patients with high VEGF-A and PDGFR-β expression levels in tumor cells and high CD31 expression levels in the tumor vasculature were more likely to response from anti-angiogenic treatment [17]; and 3) increased plasma levels of vascular cell adhesion molecule-1, decreased plasma levels of E-selectin [18], and high baseline levels of p53, HER2, and tumor apoptosis in tumor cells were correlated with a poor clinical response [19].

Current therapies, including bevacizumab (Avastin; Genentech, Inc., San Francisco, CA) [15-19], have had minimal effects on overall survival in IBC patients because of our poor knowledge of IBC’s biologic characteristics and of its specific prognostic markers. Abnormal mRNA VEGF levels [3,6,14] and high circulating VEGF levels [20] are more often associated with IBC than with non-IBC. However, the precise localization of VEGF-A protein (epithelial tumor cells and tumor stromal components) and its role as a prognostic marker in IBC tumors remain unknown. Given the known role of host factors in anti-VEGF-A resistance [21] and the stroma’s influence on cancer phenotype and aggressiveness and on patient outcome [22], we determined the protein expression of VEGF-A, VEGF-R1, and VEGF-R2 in a large set of IBC cases and correlated this expression level with known biomarkers, lymph node (LN) status, endocrine treatment, and breast cancer-specific (BCSS) and disease-free survival (DFS) duration.

## Methods

### Patients and specimens

IBC was clinically defined by a rapid onset (i.e., clinical evolution of less than 6 months) of breast edema and erythema, peau d’orange, warmth, and with or without underlying mass, and a histological confirmation of invasive breast carcinoma, with or without evidence of dermal lymphatic invasion. Tumors were histologically graded according to the Scarff-Bloom-Richardson classification system [23]. We identified 117 patients with stage IIIB IBC who had been treated at the Pierre et Marie Curie Cancer Center (PMCCC) (Algiers, Algeria) from August 2005 to March 2009. We obtained formalin-fixed, paraffin-embedded surgical incisional biopsy specimens that had been collected before any systemic treatment and normal breast tissues from 25 reduction mammoplasty patients. Normal breast tissue in 16 cases was derived from reduction mammoplasties, and normal tissue in the remaining 9 cases was taken at least 2 cm from the primary IBC tumor. We used tissue samples to build tissue microarrays (TMAs). In brief, hematoxylin-stained slides were used to delineate the tumor region on the donor block, and two 1.5-mm cores were obtained from each tumor sample using the advanced tissue arrayer (Millipore, Billerica, MA). The baseline demographic and clinical-pathological information and estrogen receptor (ER), progesterone receptor (PR), HER2, and epidermal growth factor receptor (EGFR) expression levels have been previously described [24]. This observational study was performed on anonymous paraffin blocks and was approved by the University of New Mexico Cancer Center and PMCCC Institutional Review Boards with a waiver for patient’s consent due to the retrospective nature of the study.

### Evaluation of VEGF-A, VEGF-R1, and VEGF-R2 expression

Immunohistochemical staining for VEGF-A, VEGF-R1, and VEGF-R2 was performed using validated antibodies prior to being performed in tumor sections, as we have previously described [25]. In brief, 5-μm formalin-fixed, paraffin-embedded sections were cut from tissue microarrays, placed on SuperFrost/Plus slides (Fisher Scientific; Fair Lawn, NJ), and dried for 1 hour at 60°C. When only limited tumor samples existed in the two 1.5-mm TMA cores or the core tissue had been lost during the immunohistochemical procedure, a full face section from the original block was used (10 cases for VEGF-R1 and 3 for VEGF-R2, or 10% and 3% of samples, respectively). Sections were deparaffinized in xylene and rehydrated through graded alcohols to water. Antigen retrieval (Diva solution, Biocare; Concord, CA) was performed for all antibodies at 95°C for 20 minutes in a decloaking chamber (Biocare), followed by incubation for 20 minutes in 3% hydrogen peroxide in phosphate buffer solution (1x; Invitrogen; Carlsbad, CA) to block endogenous peroxidase activity. Endogenous biotin was blocked by incubation for 10 minutes with Background Sniper (Biocare). To block non-specific protein binding, sections were treated with 3% normal goat serum and 0.05% Tween-20 (Biorad; Hercules, CA) in 1x APK buffer (Ventana Medical Systems, Inc.; Tucson, AZ) for 20 minutes at ambient temperature. They were then incubated with rabbit monoclonal VEGF-A (Biocare, PME 356 AA; dilution: ready to use; for 1 hour at ambient temperature), rabbit polyclonal VEGF-R1 (Abcam, Ab2350; dilution: 1:50; for 1 hour at ambient temperature), and rabbit polyclonal VEGF-R2 (Abcam; Ab2349; dilution: 1:200; at 4°C; overnight) antibodies. Detection was carried out using the MACH4-HRP polymer detection kit (Biocare), following the manufacturer’s instructions. Angiosarcoma tissue that was previously found to be positive for VEGF-A, VEGF-R1, and VEGF-R2 was used as a positive control; the same tissue, incubated with an isotypic-matched antibody, was used as the negative control. Sections were lightly counterstained with hematoxylin, dehydrated in graded alcohols, cleared in xylene, and coverslipped. Images were acquired from TMA cores or full face slides and digitized using the Aperio System (Vista, CA).

### Scoring of VEGF-A, VEGF-R1, and VEGF-R2 expression

VEGF-A, VEGF-R1 (cytoplasmic), and VEGF-R2 (cytoplasmic and nuclear) staining was scored for tumor and normal epithelial cells using an H-score that had been obtained by multiplying the staining intensity (graded as 0, negative; 1+, weak; 2+, moderate; and 3+, strong) by the percentage of epithelial tumor cells with positive cytoplasmic or nuclear staining (0% to 100%). Stromal cell staining was scored as 0, negative; 1+, weak; 2+ moderate; and 3+, strong. Scoring on digitized images was performed by a pathologist (Y.G.) who was blinded to all clinical data, including treatment and patient outcome. Because tumors may have abnormal protein expression (upregulation or downregulation), we determined VEGF-A, VEGF-R1, and VEGF-R2 epithelial and stromal expression levels in normal, non-neoplastic specimens and compared them with those in IBC specimens. The median value of the H-scores in normal breast biopsy samples was selected as the cut-off. For statistical analysis, epithelial cells were grouped into low- (H ≤median) or high- (H >median) expressing populations, and the expression level in stromal cells was defined as low (0-1+) and high (2+−3+).

### Statistical analysis

The primary endpoint for this study was the association between the expression of the three biomarkers and BCSS and DFS; as a secondary endpoint, we compared this expression with patients’ responses to endocrine therapy. BCSS was calculated from the date of diagnosis, with death scored as an event and censoring at the date of last follow-up or non-disease-related death. The DFS interval was calculated from the date of mastectomy to the development of first recurrence (any recurrence, local or distant). Patients without recurrence were censored at the time of last follow-up or death. Chi-square and Fisher’s exact tests were used to compare demographic and clinical-pathological data. The Spearman test was used to determine the association between the expression status of biomarkers (i.e., ER, PR, EGFR, HER2, VEGF-A, VEGF-R1, and VEGF-R2). BCSS and DFS, defined by biomarker status and other variables (age, LN status, tumor grade, chemotherapy, radiotherapy, and hormone therapy), were plotted using Kaplan–Meier curves and compared using the log-rank test. Variables found to be statistically significant in the univariate analyses were included in a step-wise (multivariate) Cox model. The multivariate models obtained for BCSS and DFS were verified by subset analysis and backward elimination [26]. The Cox model results were reported with hazard ratios (HRs) and associated 95% confidence intervals (CIs). Two-tailed P values less than 0.05 were considered statistically significant. The SAS 9.3 (SAS Inc, Cary, NC) statistical software package we used for the statistical analysis.

## Results

### Clinical-pathological data

We identified 117 stage IIIB IBC cases (103 ductal, 8 lobular, 2 metaplastic, 3 micropapillary and 1 papillary IBC) and 25 normal breast tissues who had been treated at the PMCCC (Algiers, Algeria) [24]. For this study, we evaluated 103 ductal IBC tissue samples. All IBC patients had undergone neoadjuvant anthracycline-based chemotherapy. Mastectomy was performed in 93% of patients, and the remaining 7% died of disease before surgery could be performed. Radiation therapy was administered in 85% of patients. Adjuvant endocrine therapy for ER+ patients consisted of tamoxifen and goserelin in premenopausal women (56%) and aromatase inhibitors in postmenopausal women (44%).

### VEGF-A, VEGF-R1, and VEGF-R2 protein expression in normal and IBC samples

**Figure 1:**
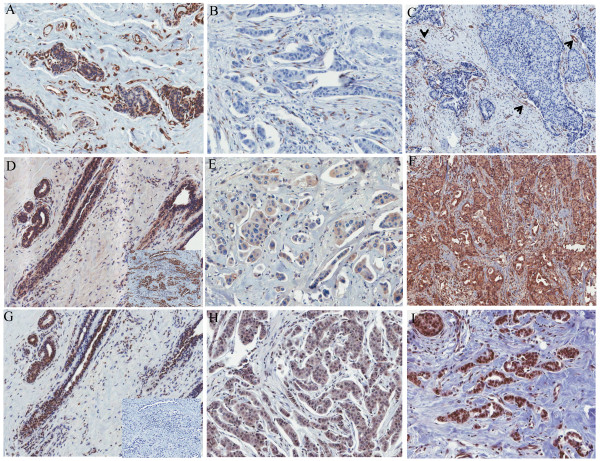
**Representative staining of VEGF-A (A-C), VEGF-R1 (D-F), and VEGF-R2 (G-I) in IBC and normal breast (A, D, G) samples.** With the H-score, IBC49 (B) expressed no epithelial VEGF-A but had low expression (1+) of stromal VEGF-A; IBC65 (C) was also negative for epithelial VEGF-A but had high expression (3+) of stromal VEGF-A (indicated by arrows). IBC samples with low (E, H) and high (F-I) VEGF-R1 (E, F) and VEGF-R2 (H, I) expression, respectively. An angiosarcoma was used as the positive (inset in D) and negative control (isotype-matched antibody; inset in G). Slides were scanned and digitized using the Aperio digital system (Vista, CA).

VEGF-A, VEGF-R1, and VEGF-R2 immunoreactivity was observed in normal breast epithelial cells, underlying luminal epithelial cells, vascular endothelial cells, and stromal fibroblasts (Figure 1A, D, and G). We found significantly (P <0.01) lower cytoplasmic VEGF-A expression levels in IBC tumor epithelial cells than in normal breast tissues (Figure 1B and C), cytoplasmic VEGF-R1 expression levels were slightly higher (Figure 1E and F; P = 0.25), and cytoplasmic VEGF-R2 expression levels were significantly higher (Figure 1H and I; P = 0.04)(Figure 1, Table 1 and Additional file 1: Figure S1: VEGF-A, VEGF-R1, and VEGF-R2 protein expression in normal and IBC samples). We also noted significant variations in VEGF-A levels in the tumor stromal tissue, with low and high expression noted in 37.9% and 62.1% of tumors, respectively (Figure 1, Table 1). VEGF-A expression in tumor stromal elements varied, indicating that stromal VEGF-A levels are correlated with different tumor biologic behaviors (see Additional file 2: Table S1: Stromal staining in normal and IBC cases). Representative examples of tumors with low and high VEGF-A stromal expression levels are shown in Figure 1B and C.

**Table 1:** VEGF-A, VEGF-R1, and VEGF-R2 expression in normal and IBC cases

**Variable**	**Normal cases (%)**	**IBC cases (%)**	P**
**VEGF-Ac**^**(21,90)**^			
Low expression	11 (52.4)	89 (98.9)	
High expression	10 (47.6)	1 (1.1)	<0.01
**VEGF-R1c**^**(25,96)**^			
Low expression	13 (52.0)	36 (37.5)	
High expression	12 (48.0)	60 (52.5)	0.25
VEGF-R2c*^(20,97)^			
Low expression	12 (60.0)	32 (33.0)	
High expression	8 (40.0)	65 (67.0)	0.04
**VEGF-R2n**^**(20,97)**^			
Low expression	11 (55.0)	48 (49.5)	
High expression	9 (45.0)	49 (50.5)	0.81
**VEGF-As**^**(20,103)**^			
Low expression	0	39 (37.9)	
High expression	20 (100)	64 (62.1)	<0.01
**VEGF-R1s**^**(25,96)**^			
Low expression	14 (56.0)	38 (39.6)	
High expression	11 (44.0)	58 (60.4)	0.18
**VEGF-R2s**^**(20,100)**^			
Low expression	6 (30.0)	48 (48.0)	
High expression	14 (70.0)	52 (52.0)	0.22

* Letter c stands for cytoplasmic, n, nuclear and s, stromal. The numbers of evaluated normal and IBC cases for each variable are shown as superscript in parenthesis; **, Overall P value using Fisher’s exact test. In some cases the tumor epithelial cells were lost during the immunoassay but the stromal component remained and was scored. This explains the discrepancies between the number of cases assessed for the epithelial and stromal contents.

### Relationship between tumor stromal VEGF-A expression and biomarker status and clinical-pathological features

Of the clinical-pathological variables (age, size, LN, and tumor grade) and biomarkers (ER, PR, HER2, EGFR, TN status, VEGF-R1, and VEGF-R2) analyzed, tumor stromal VEGF-A expression levels were strongly correlated only with both epithelial and tumor stromal VEGF-R1 levels (P = 0.03 for both).

### Tumor stromal VEGF-A and patient outcome

**Figure 2:**
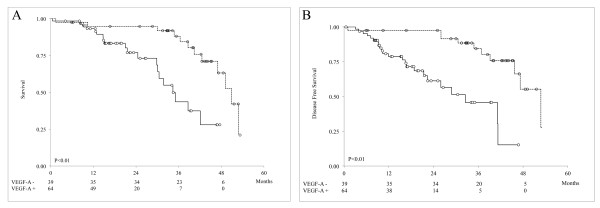
**Kaplan-Meier survival estimates of BCSS (A) and DFS (B) in IBC patients with low (dotted line) and high (continuous line) tumor stromal VEGF-A**. The numbers of patients at risk of death from IBC are shown at 12, 24, 36, and 48 months below the x axis.

Tumor stromal VEGF-A expression was a strong prognostic marker for both BCSS and DFS, as determined by Kaplan-Meier analysis (P <0.01 for both, long-rank test; Figure 2A, B). Because most IBC patients were negative for epithelial VEGF-A, it was not feasible to assess its value as a prognostic marker of patient outcome by Kaplan-Meier analysis. These findings suggest that tumor stromal VEGF-A is of significant utility in predicting clinical outcome in IBC patients.

### Tumor stromal VEGF-A status in patients with ER, PR, HER2, and TN tumors

**Figure 3:**
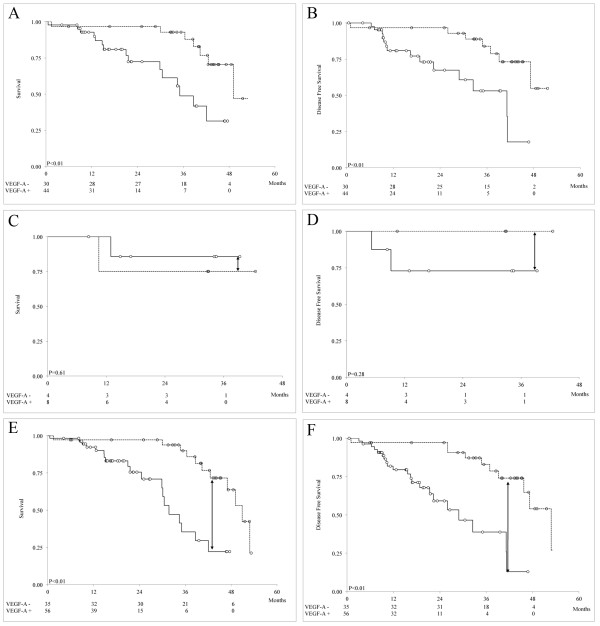
**Kaplan-Meier survival estimates of BCSS (A, C, E) and DFS (B, D, F) in ER+ (A, B), LN- (C, D), and LN+ (E, F) IBC patients with low (dotted line) and high (continuous line) stromal VEGF-A**. At month 39, a 3.9-fold reduction in BCSS (indicated by arrow in 3E), and 5.6-fold reduction and DFS (indicated by arrow in 3F) was observed in LN+ patients. The numbers of patients at risk of death from IBC are shown at 12, 24, 36, and 48 months below the x axis.

Given the prognostic and predictive value of ER, PR, and HER2 status for stratifying patients for treatment, we determined whether tumor stromal VEGF-A expression is also a strong prognostic marker in ER+, PR+, HER2+, and TN patients. A Kaplan-Meier survival analysis demonstrated that high tumor stromal VEGF-A expression levels were an important prognostic factor for poor BCSS in ER+ (Figure 3A; P <0.01) and HER2+ patients (see Additional file 3: Figure S2: Kaplan-Meier survival estimates of BCSS and DFS in IBC patients positive for PR (A, B) and HER2 (C, D); P = 0.04) and of poor DFS, regardless of ER (Figure 3B; P <0.01), PR, or HER2 status (see Additional file 3: Figure S2B, D; P = 0.03 for both). Therefore, tumor stromal VEGF-A expression appears to be a predictor of clinical outcome that is independent of these well-known epithelial markers.

In patients with TN tumors, which are generally poorly differentiated and are associated with a poor clinical outcome [27], we found that high tumor stromal VEGF-A was marginally associated with poor BCSS (P = 0.05) but not with DFS (P = 0.15).

### Tumor stromal VEGF-A status in LN- and LN+ patients

In clinical practice, the only factor that has consistently been used to determine whether patients require aggressive systemic therapy is LN status, and it is often used as a critical predictor of disease recurrence, metastasis, and survival in breast cancer patients [28]. As illustrated in Figure 3, high tumor stromal VEGF-A expression was not associated with poor BCSS and DFS in LN- patients (Figure 3C and D), but it was strongly associated in LN+ patients (Figure 3E and F; P <0.01 for both). Of note, at month 39, there was a 3.9- and 5.6-fold reduction in BCSS and DFS, respectively, in LN+ patients with high stromal VEGF-A expression levels (compare Figure 3C with 3E and 3D with 3F). Given this strong association with patient outcome, tumor stromal VEGF-A expression may be useful for identifying patients with LN+ tumors who require early interventions and more aggressive therapies.

### Tumor stromal VEGF-A status and endocrine treatment

**Figure 4:**
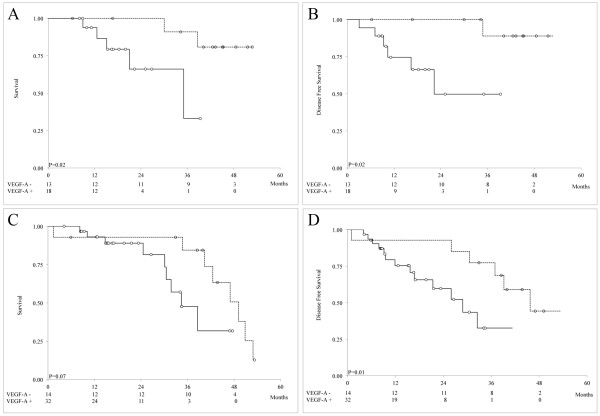
**Kaplan-Meier survival estimates of BCSS (A, C) and DFS (B, D) in IBC patients treated with tamoxifen (A, B) but not endocrine therapy (C, D) with low (dotted line) and high (continuous line) stromal VEGF-A.** The numbers of patients at risk of death from IBC are shown at 12, 24, 36 and 48 months below the x axis.

In a subset analysis of endocrine treatment, high tumor stromal VEGF-A was found to be a strong predictor of poor BCSS in patients receiving tamoxifen (Figure 4A; P = 0.02) but not in patients receiving aromatase inhibitors (see Additional file 4: Figure S3A: Kaplan-Meier survival estimates of BCSS and DFS in IBC patients treated with aromatase inhibitors with low and high stromal VEGF-A expression levels; P = 0.07) and patients who did not undergo endocrine therapy because of negative hormone receptor status (Figure 4C; P = 0.07). High tumor stromal VEGF-A levels were also a strong predictor of poor DFS in patients who received tamoxifen (Figure 4B; P = 0.02) compared with in patients who received aromatase inhibitors (see Additional file 4: Figure S3B; P = 0.11). These findings suggest that tumor stromal VEGF-A expression is associated with tamoxifen but not aromatase inhibitor resistance. Of note, high VEGF-A levels were also predictive of poor DFS in patients who did not undergo endocrine therapy (Figure 4D; P = 0.01). If the natural history of IBC proceeds as it did in patients not treated with endocrine therapy (Figure 4D), then tamoxifen may have exerted a protective effect in patients with high stromal VEGF-A levels; these patients experienced no relapses from 22 to 40 months (see Additional file 4: Figure S3C), whereas patients who were not provided endocrine therapy experienced a steady rate of relapses during the same period. The difference between these two groups was not significant (P = 0.86), although this is a hypothetical comparison given the molecular differences between the two populations (see the Discussion section).

### Multivariate analyses

We used a Cox proportional hazards model, with death from breast cancer (median BCSS, 25.1 [range, 0.5-53.2 months]) and time to recurrence (median DFS, 21.5 months [range, 0.5-53.2 months]) as the endpoints and tumor grade, LN status, radiotherapy and hormone treatment, ER/PR, HER2, EGFR, TN, VEGF-R1, VEGF-R2, tumor stromal VEGF-A, and tumor stromal VEGF-R1 as the predictive variables. Using a stepwise evaluation, verified by backward and subset variable analyses, we determined that tumor stromal VEGF-A expression was the best predictor tested (Table 2). Axillary LN involvement at presentation was noted in 88% of IBC patients; however, it was not significant on multivariate analysis. Tumor grade and hormonal treatment were not associated with DFS (P = 0.06 for both). The significant predictors of BCSS and DFS were tumor stromal VEGF-A and HER2 and tumor stromal VEGF, respectively, with tumor stromal VEGF-A being the strongest predictor of poor BCSS (HR, 5.0; 95% CI: 2.0-12.3; P <0.01) and DFS (HR, 4.2; 95% CI: 1.7-10.3; P <0.01).

**Table 2:** Multivariate Cox proportional hazard model

	BCSS*		**Disease Free Survival**	
**Variable**	**HR (95% CI)**	**P value**	**HR (95% CI)**	**P value**
VEGF-As^(101,103)^	5.0 (2.03-12.3)	<0.01	4.2 (1.7-10.3)	<0.01
HER2^(101, 103)^	2.7 (1.07-6.8)	0.04		NS
Hormone treatment^(101,103)^		NS	0.5 (0.25-1.0)	0.06
Tumor grade^(101,103)^		NS	2.1 (0.96-4.5)	0.06

* BCSS, breast cancer-specific survival; HR, hazard ratios; CI, confident intervals; VEGF-As, tumor stromal VEGF-A; NS, not significant. The numbers of evaluated IBC cases are shown as superscript in parenthesis.

## Discussion

Bevacizumab binds to VEGF-A, blocking its biological activity, which in turn affects the vasculature that supports tumor growth [12,29]. The biological rationale behind bevacizumab use in clinical trials is that tumor VEGF-A expression levels will determine response to bevacizumab treatment. Clinical trials of bevacizumab in breast cancer, including IBC, have demonstrated that patients with high basal tumor VEGF-A expression levels experience a response [17], but VEGF-A expression is not predictive of outcome [16,18,19]. In our study, we found that tumor stromal VEGF-A expression levels were a strong independent predictor of BCSS and DFS in IBC patients; that the tumor stromal VEGF-A level is predictive of DFS, regardless of ER, PR, HER2, and LN status; and that treatment response to tamoxifen (not to aromatase inhibitors) is associated with the tumor stromal VEGF-A expression level.

Axillary LN involvement at presentation is noted in about 55% to 85% of patients with IBC, and LN status remains an important prognostic indicator [1,28]. However, LN was not significant in the multivariate analysis. Similarly, in a previous study, no significant association was found between overall survival and disease-specific survival rates and LN status in IBC patients [30]. Although these findings are of considerable interest and may explain the lack of correlation between bevacizumab treatment and VEGF-A expression, the data must be interpreted with caution. IBC is a rare disease; to our knowledge, the current study is the largest analysis of VEGF-A, VEGF-R1, and VEGF-R2 expression in IBC. However, as we previously noted [24], our research has the drawbacks inherent to retrospective studies [31]; therefore, these findings warrant further independent confirmation.

Various tumor models [32,33], including IBC [22], have been used to demonstrate that the supportive network provided by the stroma is critical to a cancer’s phenotype and aggressiveness and to patient outcome. Although the cause of high VEGF-A expression levels in the breast tumor stroma is unknown, a significant increase in human VEGF-A levels in the serum and tumor was observed in the WIBC-9 murine xenograft, along with a significant increase in murine VEGF-A levels [20]. Furthermore, hypoxia, a major inducer of VEGF in tumors and a characteristic feature of IBC [34], induces upregulation of VEGF in mammary fibroblasts [35]. This confirms the known compensatory upregulation of host VEGF-A [21]; on the other hand, it emphasizes the need to completely block VEGF-A to achieve maximal tumor growth inhibition [12,21]. Our data support the theory that higher doses of bevacizumab are needed in IBC patients to completely block high tumor stromal VEGF-A expression levels and achieve optimal tumor inhibition. However, this may be clinically impossible given the observed toxic adverse events that result from the doses currently in use [36,37]. Because of the observed co-expression of VEGF-A and other angiogenic factors, additional targeting of other signaling pathways is needed to achieve optimal clinical responses. Higher levels of angiogenic factors, such as thromboxane A2 receptor, cyclooxygenase-2, angiopoietin 2, and thrombomodulin, and chemokines, such as stromal-derived factor 1 and its receptor CXCR-4, have been reported in IBC than in non-IBC patients [6,38,39]. These factors, alone or in combination with VEGF-A, may promote IBC’s metastatic potential. In particular, CXCR-4, which is associated with brain metastases in IBC [40], is stimulated by VEGF-A [41], linking VEGF-A expression to the migratory potential of tumor cells. These molecules may also be good candidates for theranostic applications, in combination with anti-angiogenic treatments.

In a subset analysis of the efficacy of endocrine therapy response in IBC patients, a high tumor stromal VEGF-A expression level was significantly associated with both poor BCSS and DFS in tamoxifen-treated patients. Interestingly, tumor stromal VEGF-A expression was also significantly associated with poor DFS in patients who did not undergo endocrine therapy. It is impossible to draw a definitive conclusion about the role of tumor stromal VEGF-A and tamoxifen treatment because of the lack of a subset of ER+ patients who did not receive tamoxifen because of ethical considerations; however, we considered patients not undergoing endocrine therapy because of negative ER status as an indicator of the natural course of the disease. Tamoxifen exerted a protective effect, as demonstrated by the absence of DFS events from months 22 to 40; during the same period, a continuous decrease in survival duration was observed in patients who did not undergo endocrine therapy (see Additional file 4: Figure S3C). However, the two groups differed molecularly. Further studies are needed to determine whether stromal VEGF-A is an indicator of tamoxifen resistance.

As for the mechanisms that implicate VEGF-A in tamoxifen response, reactive stroma and vessels may produce growth factors that stimulate tumor cells such that tumor’s inhibitory effect on tumor growth is bypassed by paracrine tumor growth stimulatory pathways, resulting in high angiogenesis with hormone resistance [42]. In addition, tumor cells, under tamoxifen pressure, may produce growth factors that directly or indirectly stimulate angiogenesis. Specifically, tamoxifen induces an increase in tumor growth factor β1 expression in tumor cancer cells and stromal fibroblasts [43,44], which in turn, can increase VEGF-A expression in both breast tumor cells and tumor-associated macrophages [45,46]. This VEGF-A release by activated stroma may increase the growth of ER+ malignant epithelial cells and adjacent normal epithelium [47]. These findings and our data indicate that IBC patients with high tumor stromal VEGF-A levels will not benefit from tamoxifen but may benefit from a combination of tamoxifen and anti-angiogenic treatment.

## Conclusions

In this study, tumor stromal VEGF-A expression was associated with an increased risk of breast cancer death and recurrence in IBC patients, independent of clinical-pathological risk factors and tamoxifen treatment. Tumor stromal VEGF-A expression levels at diagnosis may be an effective prognostic factor that will allow individualization of therapy. In future prospective clinical trials, the prognostic power of tumor stromal VEGF-A expression should be confirmed in IBC patients.

## Competing interests

The authors declare that they have no competing interests.

## Authors’ contributions

H.A.P. participated in the conception, design, and analysis of data and wrote the manuscript with input from all authors; N.C. participated in retrieving cases, performing chart reviews, and analyzing the data; Y.G. participated in immunostain scoring and data interpretation and drafted the manuscript; C.Q. performed the statistical analysis; J.V. carried out immunoassays and data interpretation; M.R. participated in study design and coordination and data interpretation and helped draft the manuscript. All authors read and approved the final manuscript.

## Pre-publication history

The pre-publication history for this paper can be accessed here:

http://www.biomedcentral.com/1471-2407/12/298/prepub

## Supplementary Material

Additional file 1 **Figure S1.** VEGF-A, VEGF-R1, and VEGF-R2 protein expression in normal (N) and IBC (I) samples. H-scores were taken as continuous variables and plotted as relative units (RU). Significant differences are indicated by a horizontal line with the corresponding P value (unpaired t-tests). The letter c stands for cytoplasmic and n for nuclear. The median H-score for normal tissues were taken as the cut-off. VEGF-A: median, 80 (SD, 52.5; range, 0–200); VEGF-R1: median, 127.5 (SD, 89.8; range, 0–300); VEGF-R2 cytoplasmic: median, 90 (SD, 86.3; range, 0–300); VEGF-R2 nuclear: median, 60 (SD, 55.4; range, 0–180). (DOCX 32 kb) Click here for file

Additional file 2 **Table S1.** Stromal staining in normal and IBC cases. (TIFF 2741 kb) Click here for file

Additional file 3 **Figure S2.** Kaplan-Meier survival estimates of BCSS (A, C) and DFS (B, D) in IBC patients who were positive for PR (A, B) and HER2 (C, D), with low (dotted line) and high (continuous line) stromal VEGF-A levels. The numbers of patients at risk of death from IBC are shown at 12, 24, 36, and 48 months below the x axis. (TIFF 2911 kb) Click here for file

Additional file 4 **Figure S3.** Kaplan-Meier survival estimates of BCSS (A) and DFS (B) in IBC patients treated with aromatase inhibitors, with low (dotted line) and high (continuous line) stromal VEGF-A levels. Figure 3C shows the DFS survival analysis of tumor stromal VEGF-A+ patients treated with tamoxifen (solid) and patients who did not receive endocrine therapy. The numbers of patients at risk of death from IBC are shown at 12, 24, 36, and 48 months below the x axis. (TIFF 2157 kb) Click here for file
