# Supplementary material for: Tumor stromal vascular endothelial growth factor A is predictive of poor outcome in inflammatory breast cancer
Source: BMC Cancer. 2012 Jul 19;12:298. doi: 10.1186/1471-2407-12-298 (PMC3474178; doi:10.1186/1471-2407-12-298)
Supplement: Additional file 1 — Figure S1. VEGF-A, VEGF-R1, and VEGF-R2 protein expression in normal (N) and IBC (I) samples. H-scores were taken as continuous variables and plotted as relative units (RU). Significant differences are indicated by a horizontal line with the corresponding P value (unpaired t-tests). The letter c stands for cytoplasmic and n for nuclear. The median H-score for normal tissues were taken as the cut-off. VEGF-A: median, 80 (SD, 52.5; range, 0–200); VEGF-R1: median, 127.5 (SD, 89.8; range, 0–300); VEGF-R2 cytoplasmic: median, 90 (SD, 86.3; range, 0–300); VEGF-R2 nuclear: median, 60 (SD, 55.4; range, 0–180). (DOCX 32 kb) [file 1471-2407-12-298-S1.docx]

**Additional Table 1. Stromal staining in normal and IBC cases**

| **Variable** | **Normal cases (%)** | **IBC cases (%)** | **P*** |
| --- | --- | --- | --- |
| **VEGF-A** |  |  |  |
| 0 | 0 | 21 (20.4) | 0.01 |
| 1+ | 0 | 18 (17.5) |  |
| 2+ | 11 (55.0) | 41 (39.8) |  |
| 3+ | 9 (45.0) | 23 (22.3) |  |
| **Total** | 20 | 103 |  |
| **VEGF-R1** |  |  |  |
| 0 | 1 (4.0) | 7 (7.3) | 0.36 |
| 1+ | 13 (52.0) | 31 (32.3) |  |
| 2+ | 5 (20.0) | 32 (33.3) |  |
| 3+ | 6 (24.0) | 26 (27.1) |  |
| **Total** | 25 | 96 |  |
| **VEGF-R2** |  |  |  |
| 0 | 2 (10.0) | 4 (4.0) |  |
| 1+ | 4 (20.0) | 44 (44.0) |  |
| 2+ | 12 (60.0) | 26 (26.0) |  |
| 3+ | 2 (10.0) | 26 (26.0) | 0.92 |
| **Total** | 20 | 100 |  |

*, Kruskal-Wallis exact test assessing differences between normal and IBC cases.
