# Supplementary figures and images for: Tumor stromal vascular endothelial growth factor A is predictive of poor outcome in inflammatory breast cancer
Source: BMC Cancer. 2012 Jul 19;12:298. doi: 10.1186/1471-2407-12-298 (PMC3474178; doi:10.1186/1471-2407-12-298)

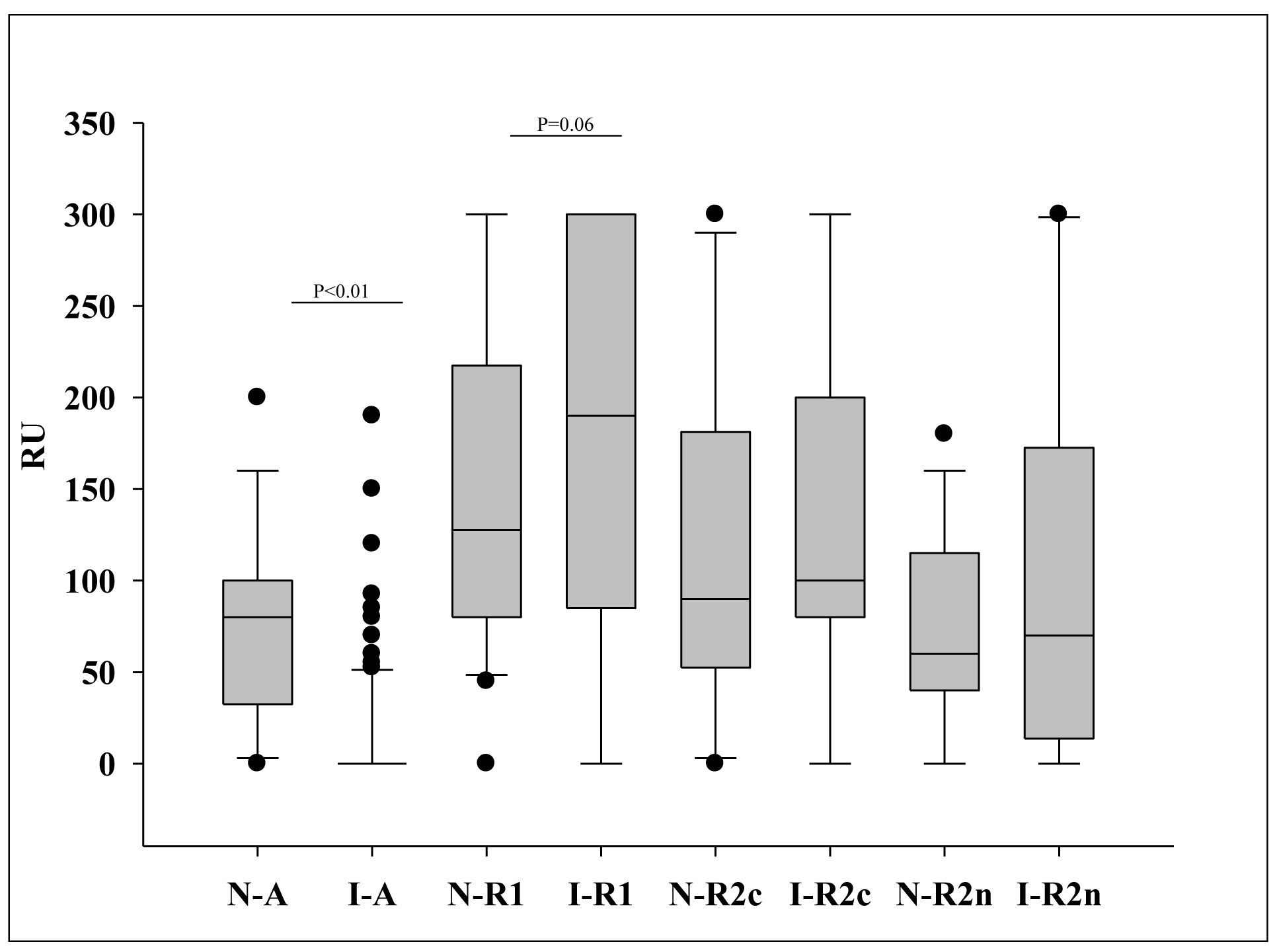

Supplement: Additional file 2 — Table S1. Stromal staining in normal and IBC cases. (TIFF 2741 kb) [file 1471-2407-12-298-S2.tiff]

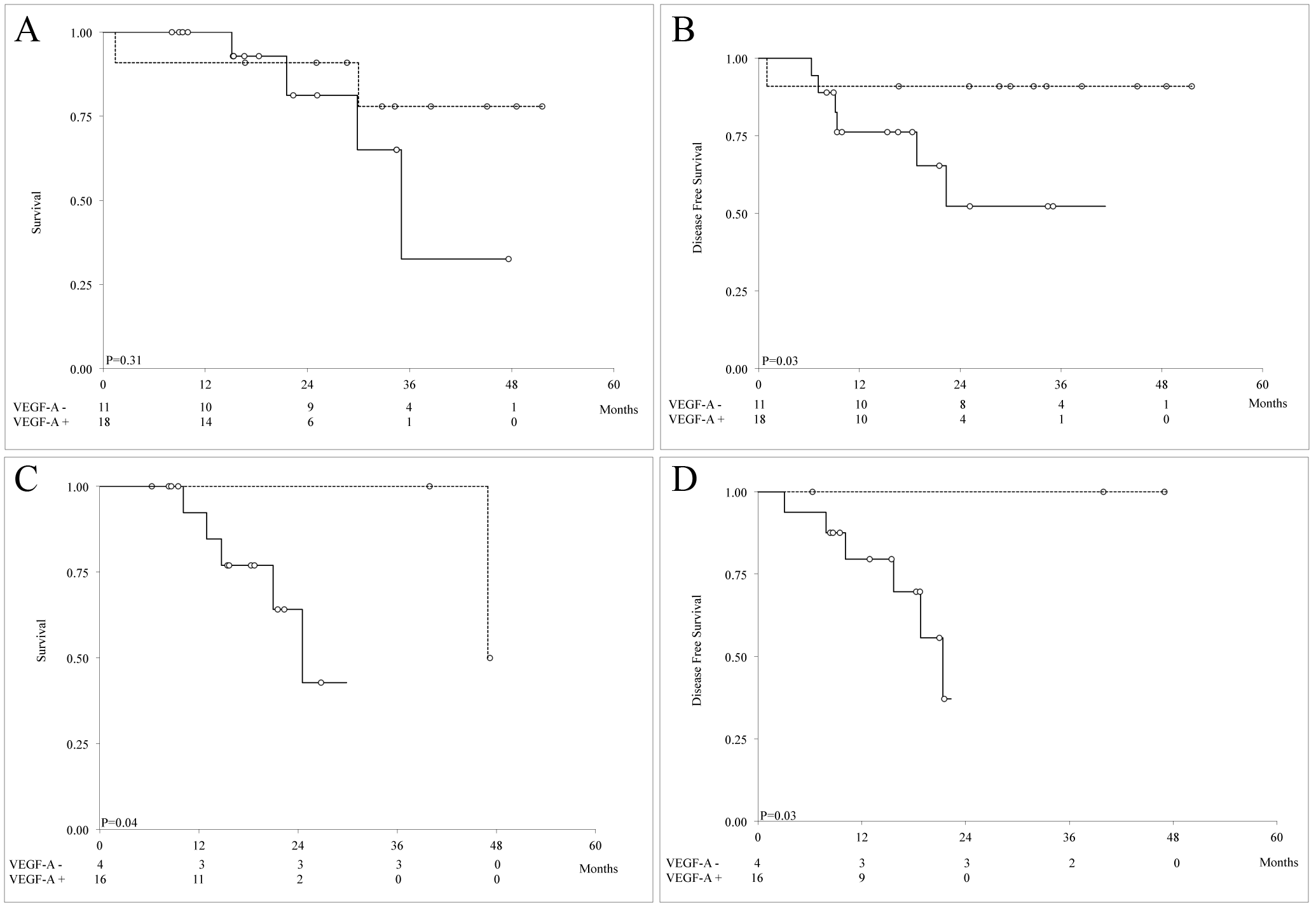

Supplement: Additional file 3 — Figure S2. Kaplan-Meier survival estimates of BCSS (A, C) and DFS (B, D) in IBC patients who were positive for PR (A, B) and HER2 (C, D), with low (dotted line) and high (continuous line) stromal VEGF-A levels. The numbers of patients at risk of death from IBC are shown at 12, 24, 36, and 48 months below the x axis. (TIFF 2911 kb) [file 1471-2407-12-298-S3.tiff]

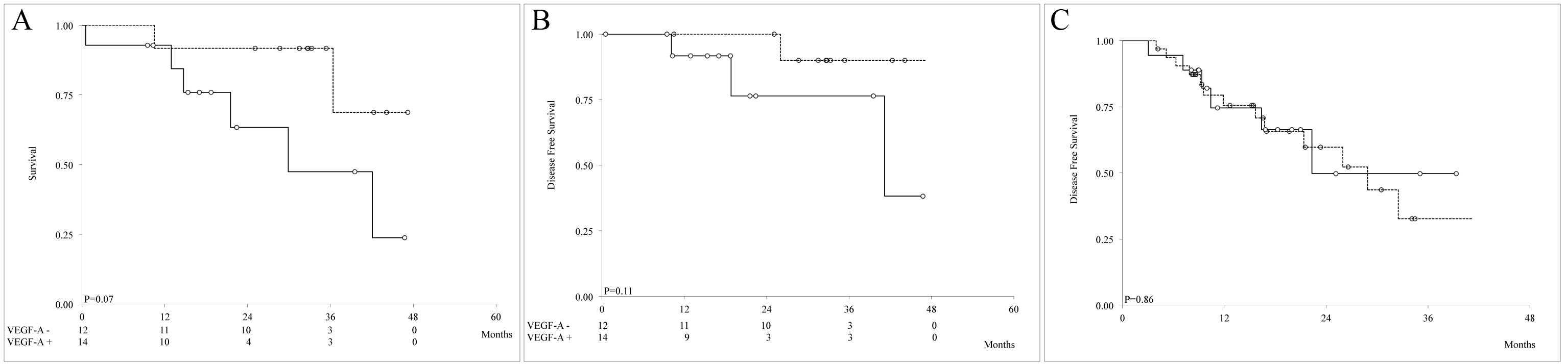

Supplement: Additional file 4 — Figure S3. Kaplan-Meier survival estimates of BCSS (A) and DFS (B) in IBC patients treated with aromatase inhibitors, with low (dotted line) and high (continuous line) stromal VEGF-A levels. Figure 3C shows the DFS survival analysis of tumor stromal VEGF-A+ patients treated with tamoxifen (solid) and patients who did not receive endocrine therapy. The numbers of patients at risk of death from IBC are shown at 12, 24, 36, and 48 months below the x axis. (TIFF 2157 kb) [file 1471-2407-12-298-S4.tiff]
